# Determining the Role of ApoE4 in Age-Related Cerebrovascular Decline

**DOI:** 10.1093/geroni/igaa057.390

**Published:** 2020-12-16

**Authors:** Kate Foley, Peter Borchian, Dylan Garceau, Kevin Kotredes, Paul Territo, Michael Sasner, Gareth Howell, Cory Diemler

**Affiliations:** 1 The Jackson Laboratory, Bar Harbor, Maine, United States; 2 Indiana University, Indianapolis, Indiana, United States

## Abstract

Cerebrovascular decline occurs during aging and may be critical during prodromal phases of Alzheimer’s disease (AD). The E4 allele of apolipoprotein E (APOE4) is the greatest genetic risk factor for AD and decreased longevity and studies suggest APOE4 increases risk for age-dependent cerebrovascular damage. To study the relationship between APOE4 and age-related cerebrovascular decline, male and female C57BL/6J (B6) mice carrying combinations of APOE alleles including APOE4 (risk) and APOE3 (neutral), as well as B6 controls were assessed at a variety of ages from 4 to 24 mos for cognitive ability, biometrics and cerebrovascular health including i) PET/MRI using 64Cu-PTSM (perfusion) and 18F-FDG (metabolism), ii) transcriptional profiling and iii) immunofluorescence. Despite no cognitive decline, male APOE4 mice showed hypo-perfusion and hypo-metabolism by 12 mos, while female APOE4 mice showed an uncoupled hyper-perfusion and hypo-metabolism phenotype. Transcriptional profiling showed differential expression of genes involved in regulation of cerebral perfusion, glucose transportation and metabolism in APOE4 mice. An age-dependent blood brain barrier compromise was also apparent in the brains of female APOE4 mice. Physical activity reduces risk for human AD and our data shows exercise improves cerebrovascular health in mice. However, the effects to cerebrovascular health in individuals carrying genetic risk factors such as APOE4 are not known. To determine whether exercise can overcome APOE4-dependent cerebrovascular damage, APOE mice are being exercised from 2-4 and to 2-12 mos. Transcriptional profiling and immunofluorescence will determine whether the benefits of exercise to the cerebrovasculature are modulated by genetic risk factors such as APOE4.

